# Effect of dexamethasone prodrug on inflamed temporomandibular joints in juvenile rats

**DOI:** 10.1186/s13075-015-0772-5

**Published:** 2015-09-24

**Authors:** Mitchell Knudsen, Matthew Bury, Callie Holwegner, Adam L. Reinhardt, Fang Yuan, Yijia Zhang, Peter Giannini, David B. Marx, Dong Wang, Richard A. Reinhardt

**Affiliations:** University of Nebraska Medical Center College of Dentistry, 4000 East Campus Loop South, Lincoln, NE 68583-0740 USA; Private Practice of Orthodontics, Cheyenne, WY USA; Pediatric Rheumatology, Children’s Hospital, Omaha, NE USA; University of Nebraska Medical Center College of Pharmacy, Omaha, NE USA; Department of Statistics, University of Nebraska, Lincoln, NE USA

## Abstract

**Introduction:**

Juvenile idiopathic arthritis (JIA) often causes inflammation of the temporomandibular joint (TMJ) and has been treated with both systemic and intra-articular steroids, with concerns about effects on growing bones. In this study, we evaluated the impact of a macromolecular prodrug of dexamethasone (P-DEX) with inflammation-targeting potential applied systemically or directly to the TMJ.

**Methods:**

Joint inflammation was initiated by injecting two doses of complete Freund’s adjuvant (CFA) at 1-month intervals into the right TMJs of 24 growing Sprague–Dawley male rats (controls on left side). Four additional rats were not manipulated. With the second CFA injection, animals received (1) 5 mg of P-DEX intra-articularly (n = 9), (2) 15 mg of P-DEX into the tail vein (n = 7), or (3) nothing in addition to CFA (n = 8). The rats were killed 28 days later and measured by radiography for ramus height (condylar superior to gonion inferior [CsGoInf]), by micro-computed tomography for condylar width (CW) and bone volume/standardized condylar volume (BV/CV), and by histology for retrodiscal inflammatory cells. Inflammation targeting of systemic P-DEX was confirmed by IVIS infrared dye imaging. Inflammation and bone growth were compared between groups using analysis of variance and Pearson’s correlations.

**Results:**

CFA caused a significant reduction in CsGoInf (*p* < 0.05), but neither route of P-DEX administration had an effect on CsGoInf or CW at CFA injection sites. BV/CV was significantly reduced in both inflamed and control condyles as a result of either steroid application (*p* < 0.05). The inflammatory infiltrate was overwhelmingly lymphocytic, comprising 16.4 ± 1.3 % of the field in CFA alone vs. <0.01 % lymphocytes in contralateral controls (*p* < 0.0001). Both P-DEX TMJ (10.1 ± 1.2 %) and systemic P-DEX (8.9 ± 1.7 %) reduced lymphocytes (*p* < 0.002). The total area of inflammatory infiltrate was significantly less in the systemic injection group than in the group that received CFA injections alone (2.6 ± 1.5 mm^2^ vs. 8.0 ± 1.3 mm^2^; *p* = 0.009), but not in the group that received intra-articular P-DEX (8.8 ± 1.2 mm^2^).

**Conclusions:**

High-dose systemic administration of inflammation-targeting P-DEX is more effective than an intra-articular injection in reducing TMJ inflammation, but both routes may affect TMJ bone density.

## Introduction

Juvenile idiopathic arthritis (JIA) is an inflammatory disease of the joints of unknown etiology. It begins before the age of 16 years and lasts more than 6 weeks at a time [1]. Magnetic resonance imaging showed that 87 % of patients with newly diagnosed JIA had temporomandibular joint (TMJ) inflammatory activity [2]. In a study in which researchers evaluated the occurrence and clinical signs and symptoms of TMJ involvement in patients diagnosed with JIA, 45 % of 97 children evaluated had TMJ involvement [3]. More recently, 55 % of patients with JIA had at least one symptomatic TMJ and 78 % of radiographed individuals had condylar lesions [4]. TMJ arthritis may cause limitations in sagittal and vertical mandibular growth, which can result in micrognathia and open bite. Many patients are required to undergo orthodontic treatment or orthognathic surgery to correct the deformities that result from the arthritic destruction of the joint and associated growth disturbances [5]. Adult rheumatoid arthritis (RA) also has a high incidence of TMJ involvement. The authors of a recent report of patients with RA showed that 57.9 % of TMJs have spontaneous pain, 87.9 % have radiologic destruction, and 42.1 % have restricted mouth opening [6].

Inflammation and pain reduction are the primary goals of pharmacologic therapy, including the use of steroids. Intra-articular injections appear to be preferred over systemic steroid applications for TMJ management in both JIA and RA [7], presumably because of more localized action and fewer systemic side effects and less impact on other bones of the skeleton [8]. In a recent systematic review, Stoustrup et al. [9] found that intra-articular TMJ corticosteroid injections are successful in reducing inflammation and orofacial symptoms in patients with JIA, but evidence is lacking regarding whether the anti-inflammatory behavior of the corticosteroids improves maximal incisal opening or normalizes and/or improves mandibular growth in these patients. In studies done in rabbits, researchers have found that intra-articular injections of corticosteroids caused a decrease in mandibular growth and that, on the basis of this evidence, intra-articular corticosteroid injections would not be appropriate in growing individuals [10]. Even in older individuals with TMJ RA, steroid injections are not useful for long-term or repeated treatment, as they lead to collapse of the joint [11]. More conservative approaches have been recommended.

Are there protocols that can concentrate steroids in the TMJ without use of intra-articular injections? One proposed option is dexamethasone iontophoresis, which used low-grade electric currents for transdermal dexamethasone delivery into the TMJ [12]. An average of eight sessions resulted in increased interincisor opening in 68 % of patients with JIA and decreased TMJ pain in 73 %. Another possibility is a macromolecular prodrug of dexamethasone (P-DEX) that can traverse highly permeable blood vessels, such as those found in inflamed tissue associated with TMJ JIA or RA.

The present study was designed to evaluate inflammatory, growth, and morphologic changes associated with P-DEX administration for juvenile rat TMJ inflammation, delivered by either intra-articular (local) or tail vein (systemic) injection. The hypothesis of this study was that a single systemic high-dose P-DEX application could reduce TMJ inflammation as effectively as local P-DEX administration.

## Methods

### Complete Freund’s adjuvant–induced inflammation

All animals were treated and housed in the University of Nebraska Medical Center (UNMC) College of Dentistry Animal Facility, and ethical approval was obtained from the UNMC Institutional Animal Care and Use Committee (IACUC 10-70-FC). Juvenile rats were chosen because they were experiencing rapid growth throughout our study, so bone changes in the condyle during TMJ inflammation and resulting from drug application could be monitored.

Complete Freund’s adjuvant (CFA; 50 μg of heat-killed *Mycobacterium tuberculosis* in paraffin oil) was injected into the TMJ to induce inflammation according to the protocol described by Harper et al. [13] and George et al. [14]. The rats received two intra-articular injections of 50 μl of CFA 4 weeks apart, at week 0 to initiate inflammation and at week 4 to boost inflammation in conjunction with administration of experimental drugs.

### Experimental groups

**Fig. 1:**
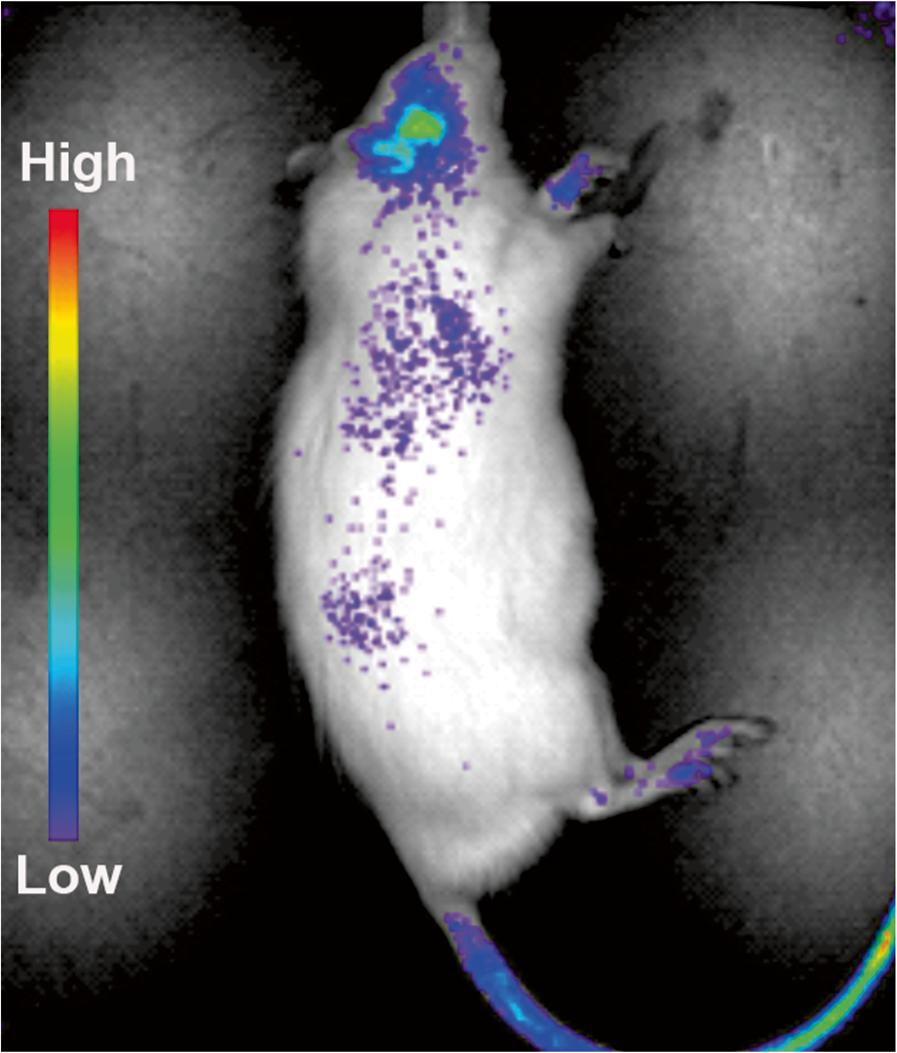
IVIS image obtained 3 days after 1 mg of prodrug of dexamethasone infrared dye was injected into the tail vein. Note intensity of color concentrated in the temporomandibular joint area

Group characteristics are summarized in Table 1. Group 1 (CFA) received 2 unilateral (right) TMJ intra-articular injections of CFA alone at week 0 and week 4. Group 2 (CFA + P-DEX TMJ) received 2 unilateral TMJ intra-articular injections of CFA (at week 0 and week 4). The week 4 intra-articular injection was combined with 5 mg of P-DEX. Group 3 (CFA + P-DEX Tail) received two unilateral TMJ intra-articular injections of CFA (week 0 and week 4) and intravenous (tail vein) injection of 15 mg P-DEX at week 4. Before the core study, 4 rats received 2 unilateral (right) TMJ intra-articular injections of CFA (week 0 and week 4) and an intravenous (tail vein) injection cocktail of a low dose of IRDye 800CW-labeled P-DEX at week 4 to confirm that P-DEX could concentrate in the inflamed TMJ (Fig. 1). Contralateral joints (left side) of injected animals served as unmanipulated controls. Previous studies have shown some inflammatory crossover effects [15]; therefore, Group 4 was included with no manipulation (both sides used). All rats were euthanized at 8 weeks.

**Table 1:** Experimental groups

Group	Animals, n	Week 0	Week 4	Week 8
1 (CFA)	8	TMJ CFA	TMJ CFA	Killed
2 (CFA + P-DEX TMJ)	9	TMJ CFA	TMJ CFA + P-DEX TMJ	Killed
3 (CFA + P-DEX tail)	7	TMJ CFA	TMJ CFA + P-DEX tail	Killed
4 (no manipulation)	4^a^	–	–	Killed

*CFA* complete Freund’s adjuvant, *P-DEX* prodrug of dexamethasone, *TMJ* temporomandibular joint

^a^Both TMJs evaluated in each animal

### Drug preparation and application

Macromolecular P-DEX was synthesized by reversible addition–fragmentation chain transfer (RAFT) copolymerization, as described previously [16]. Briefly, *N*-(2-hydroxypropyl)methacrylamide (HPMA), *N-*methacryloylglycylglycylhydrazyl dexamethasone (MA-DEX), and other comonomers (*N*-methacryloylaminopropyl fluorescein thiourea and *N*-[3-aminopropyl]methacrylamide hydrochloride[APMA]; Polysciences, Warrington, PA, USA) were copolymerized at 40 °C under argon for 48 h with 2,2′-azobisisobutyronitrile as the initiator and *S*,*S*′-bis(α,α′-dimethyl-α″-acetic acid)trithiocarbonate as the RAFT agent [17]. The resulting polymers were purified by Sephadex LH-20 column chromatography (GE Healthcare Bio-Sciences, Pittsburgh, PA, USA) and lyophilized. IRDye 800CW-labeled P-DEX (P-DEX-IRDye) was obtained via polymer-analogous reactions between poly(HPMA-*co*-MA-DEX-*co*-APMA) and *N*-hydroxysuccinimide esters of the dye [18].

### Anesthesia and killing of the rats

Anesthesia was induced by placing the rats into an incubation chamber with 1–4 % isoflurane/100 % O_2_ (1–3 L/min) and maintained with a nose cone and 0.5–2 % isoflurane/100 % O_2_ (0.5–1 L/min) during the injections. The rats were killed by CO_2_ asphyxiation. Their skulls were placed in 10 % formalin for storage before radiographic analysis, then dissected to retain the head of the condyle, fossa, and retrodiscal area for micro-computed tomography (μCT) and histologic analysis.

### IVIS IRDye measurements

P-DEX-IRDye (1 mg/rat; n = 4 rats) was injected into the tail vein and imaging in the XENOGEN IVIS-200 in vivo imaging system (PerkinElmer, Waltham, MA, USA) was performed at 1, 2, 4, 8, and 12 h after tail vein injections, then daily for 1 week. The signal intensity was quantitatively analyzed using the resident software (Living Image; PerkinElmer). This method is similar to the method used by Ren et al. [18] with a mouse model.

### Radiographic measurements

Radiographic measurements of the right and left sides of all of the rat heads were taken before μCT evaluation. The specimens were all split along the midsagittal plane. The measurements were obtained by using a digital Kodak sensor (Kodak RVG 6100; Carestream Health, Rochester, NY, USA). The sensor was fixed to a table for stability, and the cone was positioned on the mounting table so that the x-ray source, specimen, and sensor dimensions were standardized. Digital radiographs were taken of the right and left sides, with the exposure set at 0.125 seconds. Orthodontic wire 5 mm in length was used as a calibration tool in each radiograph. Radiographs were stored and measurements were obtained using MiPACS Dental Enterprise Viewer 3.1.1326 software (Medicor Imaging, Charlotte, NC, USA). The images were properly oriented by using the “protractor tool” to measure an angle of 92 degrees from the condyle to the inferior border of the ramus. From this reference angle, two points were identified: (1) condylar superior (Cs), the most superior point on the mandibular condyle; and (2) gonion inferior (GoInf), the most inferior point on the mandibular ramus. Next, CsGoInf (ramus height) was measured as the distance between the condylar superior and gonion inferior.

### Micro-computed tomography measurements

The units were scanned with a high-resolution system (SkyScan1172; Bruker microCT, Madison, WI, USA) using a method described previously [19]. All specimens were aligned on three axes (sagittal, coronal, and transaxial) until the head of the condyle from each view was centered in each frame. The coronal views designated by the SkyScan1172 software were used to obtain the measurements. Bone volume (BV) measurements were taken in the condyle in the total volume of interest, which was a 0.784 × 0.784 × 0.784-mm cube, representing the largest cube that could fit within all condyles while touching the superior border of the condyle. The cube occupied more than 50 % of the total condylar volume. The largest condylar width (CW) measurement for each condyle was also taken from the coronal views.

### Histologic measurements

Following μCT analysis, specimens were decalcified in 5 % formic acid at 4 °C for 2 weeks, blocked in a sagittal plane through the TMJ, and embedded in paraffin, and 5-μm sections were cut through the TMJ and stained with hematoxylin and eosin to reveal the head of the condyle, disc, and retrodiscal soft tissue. Using a digital microscope charge-coupled device camera and software (ProgRes; JENOPTIK, Jena, Germany), the area of inflammatory infiltrate (×100 magnification) was assessed in the entire area of the retrodiscal tissue as determined by two independent evaluators without knowledge of experimental group, cell counts were conducted in two 100-point grids (×400) in the center of the retrodiscal tissue, and all data were averaged. Also, the proper location of TMJ injections was confirmed by the appearance of CFA oil droplets in the retrodiscal soft tissue.

### Statistical analysis

Eight rats per group were assigned based on histological detection of a significant decrease in inflammation with administration of anti-inflammatory drugs in intra-articular injections [14]. Also, analysis of previous data [19] indicated that seven or eight rats would be necessary to show a statistically significant ≥10 % difference in ramus height growth among various anti-inflammatory agents, a change likely to show signs of open bite or other growth disturbances in humans requiring orthodontic therapy. Specimens were coded by animal and side (right or left) and measured by two examiners without knowledge of group designation. Each examiner repeated these measurements 2 weeks later by remeasuring 10 % of the original specimens. Analysis of variance was used for intergroup comparisons, and Pearson’s correlation coefficients were calculated to compare measurements. Repeated measures of 10 % of the specimens with a mixed-effects model repeated-measures analysis showed that the estimated variance among animals was much greater than the residual estimate (*p* < 0.0001), indicating high reproducibility [19]. Results were reported as mean ± standard deviation or change between experimental and control sides and were considered significant when *p* values were ≤0.05.

## Results

One animal died prematurely of unknown cause after administration of CFA at week 4. All other animals tolerated the procedures well. Rats in all groups ingested similar amounts of food and water, and none showed abnormal signs of pain or stress (piloerection, scratching at the jaw) beyond porphyrin staining during the first 2–3 days following TMJ injections in all groups. Uneven numbers in groups were due to inability to complete the tail vein injection in one rat (CFA + P-DEX Tail), which was switched to CFA + P-DEX TMJ.

### Radiographic measurements

**Fig. 2:**
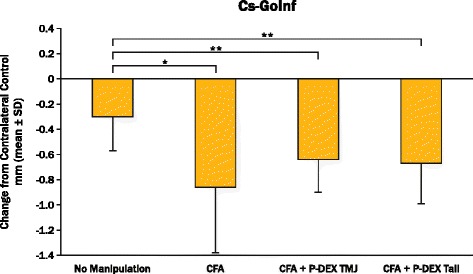
Change in ramus height for each manipulation minus its contralateral control (Cs-GoInf) 8 weeks after baseline. Brackets define statistical differences between experimental groups (sides). *p < 0.05, **p < 0.01. *CFA* complete Freund’s adjuvant, *P-DEX* prodrug of dexamethasone, *TMJ* temporomandibular joint

The radiographic data for change in ramus height from contralateral controls (CsGoInf) are shown in Fig. 2. The group with no manipulation had statistically more ramus height than all other groups, including CFA alone. Although tail or TMJ steroid injection did not cause further reduction in growth with CFA-induced inflammation, steroid application did not normalize growth. When the contralateral control mandibles were compared, those contralateral to CFA + P-DEX TMJ and CFA + P-DEX tail vein injections were statistically smaller than the ones contralateral to the no-manipulation group. However, both rami contralateral to P-DEX sides had significantly greater ramus height than the experimental sides.

### Micro-computed tomography measurements

**Fig. 3:**
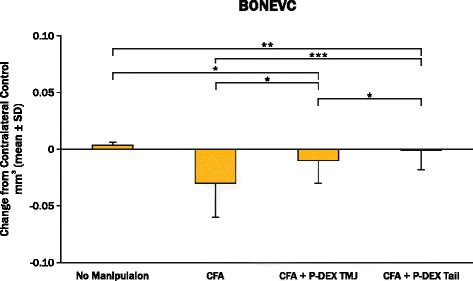
Change in condylar bone volume within standardized condylar volume of interest for each manipulation minus its contralateral control (BONEVC) 8 weeks after baseline. Brackets define statistical differences between experimental groups (sides). *p < 0.05, **p < 0.01, ***p < 0.0001. *CFA* complete Freund’s adjuvant, *P-DEX* prodrug of dexamethasone, *TMJ* temporomandibular joint

No statistically significant differences between groups or between manipulated vs. unmanipulated sides were seen for greatest CW. Differences in the bone volumetric measurement within a standardized condylar volume (BV/CV) between groups showed that the CFA + P-DEX TMJ and CFA + P-DEX tail groups had statistically less BV than the CFA alone or no-manipulation groups. Furthermore, CFA + P-DEX tail BV/CV was less than that in the CFA + P-DEX TMJ group. The contralateral controls for the CFA + P-DEX TMJ injection group and the CFA + P-DEX tail injection group had statistically less BV than the CFA contralateral control group and the no-manipulation control group. Also, the contralateral control site had statistically more BV than its CFA-alone side. However, the decrease in contralateral BV/CV resulted in little or no decrease in P-DEX BV/CV when subtracted from the experimental side (Fig. 3).

### Histologic measurements

Histologic measures of inflammation are summarized in Table 2. All experimental TMJs with CFA injections had highly significant (*p* < 0.0001) larger inflammatory infiltrates than contralateral controls (negligible infiltrate), except for the CFA + P-DEX tail group, which was not statistically different from contralateral controls. The inflammatory infiltrates were overwhelmingly lymphocytic. The percentage of lymphocytes was statistically higher in the CFA alone group vs. P-DEX TMJ or P-DEX tail group, and the P-DEX tail group had fewer lymphocytes than the P-DEX TMJ group. Plasma cells and macrophages were not present at more than 0.5 % in any tissues except CFA alone (0.7 ± 0.1 % and 0.6 ± 0.1 %, respectively). Connective tissue without infiltrate comprised the majority of retrodiscal tissue, inversely proportional to the lymphocyte infiltrate (Table 2). Whereas the CFA + P-DEX TMJ, CFA + P-DEX tail, and no-manipulation groups had more connective tissue than CFA alone, none of the three were significantly different from each other.

**Table 2:**
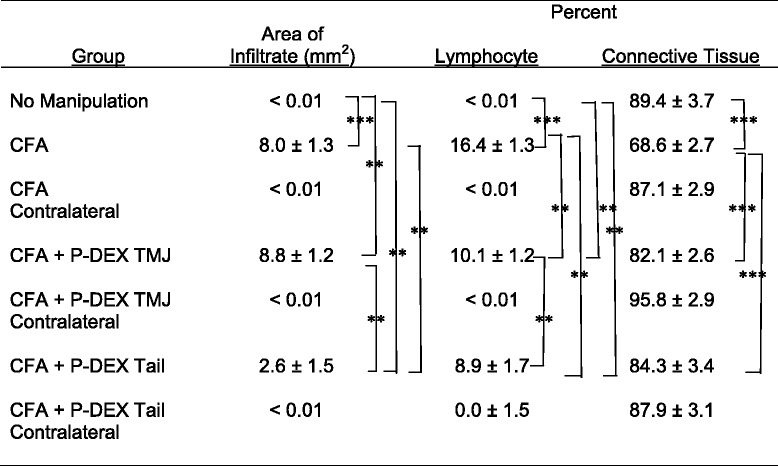
Histologic inflammation measurements in TMJ retrodiscal tissue

*CFA* complete Freund’s adjuvant, *P-DEX* prodrug of dexamethasone, *TMJ* temporomandibular joint

*p < 0.05, **p < 0.01, ***p < 0.0001

Significant correlations between ramus height and histologic levels of inflammation were found. The area of inflammatory infiltrate and percentage of lymphocytes were negatively correlated with CsGoInf (*r* = −0.31 and −0.40, respectively; *p* < 0.01), whereas the percentage of non-inflamed retrodiscal connective tissue was positively correlated with CsGoInf (*r* = 0.36, *p* = 0.004).

## Discussion

The experimental TMJ inflammation model using two CFA TMJ injections in this study was successful in sustaining inflammation for 4 weeks following the second injection (Table 2). In addition, ramus height in the CFA group was significantly smaller than in the no-manipulation group (Fig. 2) and was correlated with the markers of inflammation, as likely occurs with unpaired micrognathia and resulting deformities seen in JIA [20].

A single dose of dexamethasone, a representative steroid, was applied either locally or systemically in a P-DEX preparation with the primary goal of reducing TMJ inflammation. The systemic delivery was accomplished with the P-DEX tail group, in which the area of inflammatory infiltrate and percentage of lymphocytes were significantly reduced compared with CFA alone (Table 2). Local delivery of P-DEX TMJ also reduced the percentage of lymphocytes compared with CFA alone, but the area of infiltrate and percentage of lymphocytes were lower in the P-DEX tail group than in the P-DEX TMJ group. Although systemic steroids can be effective in reducing TMJ inflammation [21, 22], these findings suggest that systemic P-DEX was superior in reducing inflammation, probably owing to using a passive targeting mechanism termed “extravasation through leaky vasculature and the subsequent inflammatory cell-mediated sequestration (ELVIS)” [23]. Because JIA usually involves other joints in addition to TMJs, P-DEX has the advantage of selectively targeting any inflamed joint [24, 25]. The distribution of a conventional systemic steroid to the TMJ may be 1–2 % of the initial dose, far less than the 33 % differential between P-DEX TMJ and systemic P-DEX according to our experience with the adjuvant-induced arthritis (AA) rat model. The concentration of drug at the site of inflammation may help explain why patients with JIA taking conventional systemic steroids continue to have TMJ symptoms but are helped (increased maximal incisal opening and reduced arthritic changes observed by magnetic resonance imaging) by a targeted approach of steroid delivery such as intra-articular corticosteroid injections [8]. Systemic P-DEX represents another targeted approach with potentially reduced morbidity relative to intra-articular corticosteroid injections. The accumulation of P-DEX in the TMJ, using IRDye P-DEX at a lower dose (1 mg = 3 mg/kg) supports the premise that the P-DEX can leak from inflamed vessels in TMJ inflammatory arthritis through the ELVIS mechanism.

Bone quality in the condyle was affected by P-DEX via both administration routes. BV was significantly reduced by P-DEX on the CFA-inflamed side compared with no manipulation or CFA alone. Systemic steroids have traditionally been associated with reduction in BV and bone density in patients with rheumatologic diseases [26, 27], including children with JIA [28]. Local steroids also have shown disruption of TMJ bone quality in animal models. Stoustrup et al. [10], using 10-week-old female New Zealand white rabbits that had Freund’s incomplete adjuvant– or CFA-induced TMJ arthritis and that were treated with triamcinolone hexacetonide in each TMJ at four different time points or were left untreated, found that the rabbits that were treated with the corticosteroid had unfavorable mandibular growth alterations compared with the untreated arthritis and control groups. El-Hakim et al. [29] evaluated the response to intra-articular TMJ injections of dexamethasone following mechanical induction of synovitis. Histologically, they found that the condylar heads injected with dexamethasone showed resorption with active osteoclastic activity after only one injection.

Condyles contralateral to both P-DEX applications generally showed reduced BV and bone density compared with no manipulation or CFA alone. This finding confirms that systemic P-DEX was affecting bone quality in other joints, but it also indicates that P-DEX TMJ had some carryover effect on bone quality in the unmanipulated TMJ. Therefore, the present study and past results indicate that problems with bone quality associated with high-dose TMJ-targeting steroid strategies still need to be resolved. However, following JIA remission and/or discontinuation of steroids, there is clinical evidence that partial catch-up usually occurs in linear bone growth [30] and bone mineral density may return to normal in adulthood [31]. Long-term evidence in human craniofacial growth is lacking [9], but rapid remission of arthritis within the TMJ may act to eventually minimize the impact on BV and bone density loss seen in the present animal study.

P-DEX was given at high doses in the TMJ (5 mg = 15 mg/kg) and tail vein (15 mg = 45 mg/kg) based on our experience in treating AA and in the lupus nephritis mouse model (10 mg/kg and 30 mg/kg, respectively). Typical anti-inflammatory doses of human intravenous pulses of dexamethasone are 15–30 mg/kg given for 1–3 consecutive days and then continued every 2–4 weeks until response or as needed [32]. A primary benefit of intermittent pulse intravenous steroid therapy and local therapy through joint injections is the desired reduction in inflammation, but with the potential of limiting the daily exposure of steroids seen with oral systemic dosing. Daily oral steroids, when used over periods of months to years, typically result in overall doses exceeding the amount given by intermittent local or pulse intravenous doses. This daily steroid exposure is what contributes most to the increased side effects of steroids, including systemic adrenal suppression, generalized growth suppression, osteoporosis, obesity, chronic hypertension, immunosuppression, mood swings, poor sleep, cushingoid features, and avascular necrosis, further limiting their appeal for chronic use in children [32]. As a result, the chronic side effects of daily oral steroid exposure are often felt to be more harmful in the long term than the relatively rare side effects of local joint injections, such as subcutaneous soft tissue atrophy caused by steroid leakage out of the joint; localized intra-articular calcification, which rarely is clinically symptomatic; and the very rare joint infection. Similarly, the transient side effects of intermittent pulse intravenous steroid administration, such as acute hypertension, hyperglycemia, tachycardia, and acute mood swings, are usually preferable to the chronic changes seen with daily dosing. Therefore, the use of local joint injections or intermittent high-dose pulse intravenous therapy, particularly with inflammation-targeting capabilities, may be more ideal options for local TMJ arthritis, given their avoidance of daily exposure to and thereby reduction of the chronic side effects seen with daily corticosteroid exposure.

## Conclusions

Taken together, these data suggest the following. (1) Systemic application of P-DEX has the potential to concentrate in inflamed TMJs and reduce inflammation. (2) Systemic and intra-articular applications of P-DEX have no effect on ramus height or CW in growing rats with CFA-induced inflammation, but bone quality is affected. (3) TMJ inflammation appears to be associated with reduced ramus and condylar growth. (4) Further studies of systemic applications of P-DEX for treatment of TMJ inflammation, especially dosing optimizations, are warranted in both children and adults.

## Abbreviations

AA: adjuvant-induced arthritis
APMA: *N*-(3-aminopropyl)methacrylamide hydrochloride
BV: bone volume
CFA: complete Freund’s adjuvant
CsGoInf: condylar superior to gonion inferior (ramus height)
CV: standardized condylar volume
CW: condylar width
ELVIS: extravasation through leaky vasculature and the subsequent inflammatory cell-mediated sequestration
HPMA: *N*-(2-hydroxypropyl)methacrylamide
IRDye: infrared dye
JIA: juvenile idiopathic arthritis
MA-DEX: *N*-methacryloylglyclyglycylhydrazyl dexamethasone
μCT: micro-computed tomography
P-DEX: prodrug of dexamethasone
P-DEX-IRDye: prodrug of dexamethasone labeled with IRDye 800CW infrared dye
RA: rheumatoid arthritis
RAFT: reversible addition–fragmentation chain transfer
TMJ: temporomandibular joint
UNMC: University of Nebraska Medical Center
